# A Hierarchical Gamma Mixture Model-Based Method for Classification of High-Dimensional Data

**DOI:** 10.3390/e21090906

**Published:** 2019-09-18

**Authors:** Muhammad Azhar, Mark Junjie Li, Joshua Zhexue Huang

**Affiliations:** College of Computer Science and Software Engineering, Shenzhen University, Shenzhen 518060, China; azhar@szu.edu.cn (M.A.); jj.li@szu.edu.cn (M.J.L.)

**Keywords:** data mining, unsupervised classification, decision cluster, gamma mixture model, expectation maximization, high-dimensional data, curse of dimensionality

## Abstract

Data classification is an important research topic in the field of data mining. With the rapid development in social media sites and IoT devices, data have grown tremendously in volume and complexity, which has resulted in a lot of large and complex high-dimensional data. Classifying such high-dimensional complex data with a large number of classes has been a great challenge for current state-of-the-art methods. This paper presents a novel, hierarchical, gamma mixture model-based unsupervised method for classifying high-dimensional data with a large number of classes. In this method, we first partition the features of the dataset into feature strata by using k-means. Then, a set of subspace data sets is generated from the feature strata by using the stratified subspace sampling method. After that, the GMM Tree algorithm is used to identify the number of clusters and initial clusters in each subspace dataset and passing these initial cluster centers to k-means to generate base subspace clustering results. Then, the subspace clustering result is integrated into an object cluster association (OCA) matrix by using the link-based method. The ensemble clustering result is generated from the OCA matrix by the k-means algorithm with the number of clusters identified by the GMM Tree algorithm. After producing the ensemble clustering result, the dominant class label is assigned to each cluster after computing the purity. A classification is made on the object by computing the distance between the new object and the center of each cluster in the classifier, and the class label of the cluster is assigned to the new object which has the shortest distance. A series of experiments were conducted on twelve synthetic and eight real-world data sets, with different numbers of classes, features, and objects. The experimental results have shown that the new method outperforms other state-of-the-art techniques to classify data in most of the data sets.

## 1. Introduction

The classification of data is an important research topic in the field of data mining [1,2,3,4,5,6]. Intrusion detection, weather forecasting, face recognition, product recommendations, and so forth are some important applications of classification. Currently, there are several methods for classification in which hierarchical techniques are the most famous due to their clear understanding and classification accuracy. The decision tree [7,8,9] and its ensemble methods, like Bagging [10], Boosting [11], and Random Forests [12] are the most important hierarchical techniques.

In addition to pure classification techniques, clustering methods have also been used for classification purposes [13,14,15]. An early example of such methods was proposed in 1980 [16] that uses the k-means clustering method to build a cluster tree classification model. In this method, a binary cluster tree was built by interactively executing the k-means clustering algorithm. At each node, a further partition was determined by the percentage of the dominant class in the cluster node. However, only small numeric data could be classified, and every time, only two subclusters could be formed. In 2000, Huang et al. [17] proposed a new interactive approach to build a decision cluster classification model. In this approach, the k-prototypes clustering algorithm was used to partition the training data, and a visual cluster validation method [18] was adopted to verify the partitioning result at each cluster node.

With the expeditious advancement in IoT devices and social network sites [19,20], data have grown exponentially in volume and complexity, resulting in a lot of large and complex high-dimensional data. The large number of features in high-dimensional data have created the issue of the curse of dimensionality [21,22] which is characterized by correlated, sparse, noisy, and uninformative features [23]. Due to such types of complex data with a large number of classes, the existing classification techniques face a lot of challenges. Thus, the traditional decision tree performs well on a low-dimensional dataset, but in the case of high-dimensional data with multiple classes, this technique is unable to get high accuracy [24,25,26]. The main reason behind this shortcoming is how the build tree only uses a small number of features from high-dimensional data. This is because each partitioning step in building a decision tree model only considers one feature, while the information is usually stored among many features. When there is a large number of classes, overfitting occurs due to the generation of a large number of leaves [27,28,29].

To classify such high-dimensional complex data with a large number of classes, in recent years researchers have proposed several techniques. Some of these are pure classification techniques [10,11,12], while others use clustering algorithms to classify data [30,31,32,33,34]. The major issue with these techniques is the poor performance of classifying high-dimensional data with a large number of classes in terms of classification accuracy and computation cost.

To solve this key issue of classifying high-dimensional data with a large number of classes, we propose a new Hierarchical Gamma Mixture Model-based Unsupervised Method in this paper. In this hierarchical method, we apply a subspace ensemble approach to deal with this challenging problem by integrating multiple techniques in an innovative solution, named as the Stratified Subspace Sampling GMM-based Supervised method, or SSS-GMM for short. In this method, we first use the GMM Tree [35] to estimate the number of feature groups/strata given to the k-means algorithm as an input parameter. The k-means algorithm partitions the set of features of the high-dimensional dataset into a set of feature strata. After that, a stratified subspace sampling method is used to sample a set of subsets of features on feature strata by using sampling without replacement. A set of subspace data sets are generated from the set of feature subsets. These subspace data sets, produced by a stratified sampling method, are good representations of the high-dimensional data. After generating the subspace data sets, the GMM Tree algorithm is used again on each individual subspace dataset to estimate the number of clusters and initial cluster centers which are given as input parameters to the k-means algorithm to cluster the subspace dataset. Then, the link-based method [36] is used to integrate the clustering results generated from each subspace dataset into an object cluster association (OCA) matrix, on which the k-means algorithm is used to produce the ensemble clustering result with the number of clusters identified by the GMM Tree algorithm.

After producing the ensemble clustering result, the class labels are assigned to the clustering result as follows: (1) The purity of each cluster in the ensemble clustering result are computed based on the percentage of each class. (2) The dominant class label is assigned to each cluster after computing the purity and the clusters with less purity where α is discarded, and where α is the threshold set for the purity of a cluster. After assigning the class labels to the clusters, the center of each cluster is computed by finding the mean object. This clustering result with an assignment of a dominant class is used as a classifier to classify new objects as follows: (1) The distances between a new object and the center of each cluster in the classifier are computed, and the class label of the cluster is assigned to the new object which has the shortest distance.

We have conducted experiments on 12 synthetic data sets and eight real-world data sets, and compared the results of SSS-GMM with the results of k-NN, Bagging, C4.5, Random Forest, and Adaboost in terms of classification accuracy. The results show that SSS-GMM significantly outperforms all other algorithms in classifying the high-dimensional complex data with a large number of classes.

The two key contributions of this paper are:

- We integrate the multiple techniques of stratified sampling, subspace clustering, GMM Tree, k-means, and the link-based approach in an innovative algorithm to solve the challenging problem of classifying the high-dimensional complex data with the curse of dimensionality characteristics and a large number of classes.
- We report the classification accuracy by the new method on a high-dimensional complex dataset with a large number of classes and demonstrate that the new method is capable of dealing with such high-dimensional data sets. To our best knowledge, similar results were rarely reported in existing publications.

The rest of this paper is organized as follows. Related work is presented in Section 2. Section 3 reviews the GMM Tree method. Section 4 presents the SSS-GMM. Section 5 shows the experimental results on both synthetic and real-world data sets. Conclusions and future work are given in Section 6.

## 2. Related Work

In the last few decades, researchers have proposed several clustering-based classification methods to solve the supervised classification problems [13,14,16,37,38]. According to the research provided, it has been proved that classification can be considered as a high-level model in which one class can be mapped to one, or more than one cluster. Thus, classification can be considered as a clustering problem that can also be solved by the clustering techniques.

An early example of such types of methods is the interactive approach proposed by Mui et al. [16]. In this method, the k-means [39] algorithm was used to find k clusters by partitioning the training dataset without using the class label information. Then, the dominant class was found in each cluster by computing the percentage of each class by using the class label information. In the case of purity greater than 90 percent, the dominant class label was assigned to this cluster as its class. Otherwise, the cluster needed further partitioning with the k-means algorithm. After finding all clusters, the centers of each cluster was computed and a k-NN like classification model formed, in which each cluster has a dominant class and its center.

To classify the objects in the test set, the distances between the objects of the test set and the cluster centers were computed, and the class label of the cluster assigned to the new object which had the shortest distance to the new object. Since the number of clusters was much less than the number of training samples in a k-NN model, this cluster center-based classification model performed better than the k-NN model.

Zhang et al. [13] proposed a cluster-based tree algorithm to improve k-NN classification, consisting of tree construction and classification steps. This cluster-based tree algorithm performs better than the standard k-NN. Kyriakopoulou et al. [15] made another contribution to test clustering methods for classification. In their work, a new classification algorithm was proposed in which both training and testing data sets are clustered. In the next step, the dataset was augmented with meta-features, and finally, a classifier was trained on the expanded dataset. This method follows a probability mixture model in which clusters are explored based on the distribution of objects of one class in the multidimensional data space. The benefit of this method is that a classifier learns from a small training dataset by combining unsupervised learning methods with classification methods. However, a major shortcoming is that it does not perform well on high-dimensional data sets.

In 2000, Huang et al. proposed a decision clusters classifier model [17] that used the k-prototype clustering method to construct a decision clusters tree by using an interactive Fast Map algorithm. To perform classification, the k-NN-like algorithm was used. A major limitation of this method was the manual procedure of the technique, because both the building model and validation of the clusters were based on human judgment. Compared to this manual approach, the Automatic Decision Cluster Classifier (ADCC), proposed by Yan Li et al. [40], was an automatic hierarchical clustering method which used clustering algorithms in parallel to class label information during the tree construction to generate a classifier.

To improve classification accuracy, researchers have proposed several methods which generate and integrate multiple classifiers. The early work in this field has been done by Quinlan [41] and [42]. After that, ensemble classifier methods have been proposed, in which the final result could be obtained by combining the predictions of multiple classifiers. Bagging [10], Boosting [11], and random forests [12] are prominent examples of such ensemble methods which generate multiple diverse classifiers from the training dataset and use these classifiers for final classification results. Boosting uses all objects of the training data in each iteration and assigns a weight for each object based on the importance which is calculated according to the incorrectly classified objects. Thus, incorrectly classified objects will get more importance in the next iterations.

Bagging generates multiple data sets by sampling the objects with replacement from the training data. Bagging constructs classifiers from each bagged dataset. In both bagging and boosting methods, the multiple classifiers make an ensemble and are used for voting to assign the class label of the new objects. In boosting, classifiers have different vote importance, while bagging assigns the same vote for every component classifier.

As we used the clustering method to do classification, finding the number of clusters was a classical issue to do clustering. Thus, we used our GMM Tree method to find the number of clusters and the initial cluster centers, which were used to explore decision clusters. An overview of a GMM Tree is given below.

## 3. Overview of GMM Tree

This section presents an overview of the GMM Tree method [35], which plays a vital role in our proposed SSS-GMM. The GMM Tree is a hierarchical method that estimates the number of clusters and the initial cluster centers in a dataset by generating multiple levels of Gamma Mixture Models (GMMs). The estimated number of cluster and initial cluster centers are used as input parameters to the clustering algorithm to find the decision clusters in the training dataset, and these decision clusters are then used to classify the objects of the test dataset. The important steps of the GMM Tree method is discussed below.

**Figure 1:**
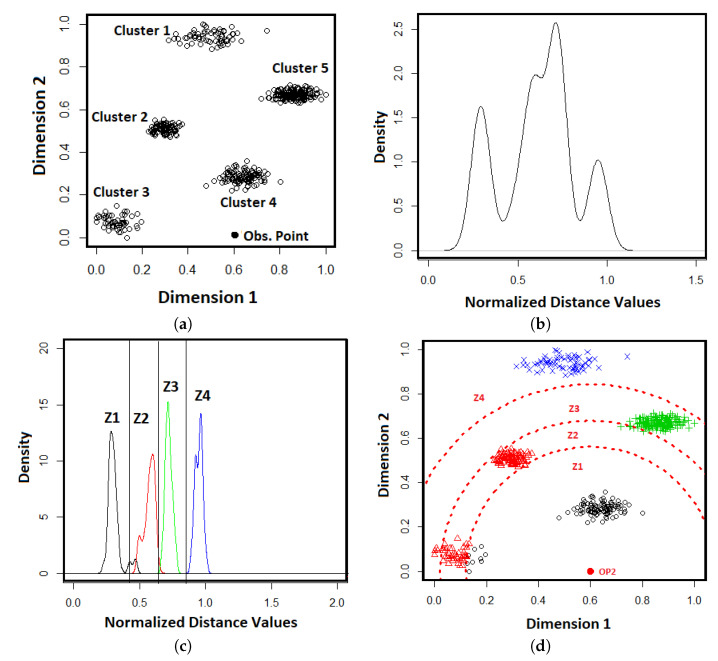
Mappings of five clusters to a distance distribution with respect to the observation point. (**a**) A sample dataset *X* in two dimensional space; (**b**) The distance distribution generated by computing the distances between the objects in the dataset *X* and the observation point; (**c**) The GMM components generated from the distance distribution; (**d**) Partition of the dataset *X* based on the GMM components generated.

Given a dataset *X* with *N* objects in which each object is a *m*-dimensional vector, the GMM Tree method allocates an observation point *p* in the data space, and the distances between the *N* objects and the allocated observation point *p* are calculated using the Euclidean distance function. This process is described in Figure 1 in which Figure 1a shows an example of dataset *X* in a two-dimensional space. The black dot (Obs. Point) is assigned as an observation point to the data space. The distribution of distances between the objects in the dataset and the observation point is shown in Figure 1b in which the peaks of the distance distribution shows the dense regions in the dataset of Figure 1a, that is, the clusters.

Let $X_{d}=\left\{x_{1},x_{2},\ldots ,x_{N}\right\}$ be a set of distance values, the distance distribution is modeled as a GMM which is defined as:

(1) $$P\left(x|\theta \right)=\sum_{\mu =1}^{M}\pi_{\mu }g\left(x|\theta_{\mu }\right),x\geq 0$$

where *M* is the number of Gamma components, $\theta_{\mu }$ are the parameters of Gamma component μ, including shape parameter $\alpha_{\mu }$ and scale parameter $\beta_{\mu }$, and $\pi_{\mu }$ is the mixing proportion of component μ. The condition $\sum_{\mu =1}^{M}\pi_{\mu }=1$ must hold to confirm that $P\left(x|\theta \right)$ is a well defined probability distribution. The probability density function of Gamma component μ is

(2) $$g\left(x|\alpha_{\mu },\beta_{\mu }\right)=\frac{x^{\alpha_{\mu }-1}}{\Gamma \left(\alpha_{\mu }\right)\beta_{\mu }^{\alpha_{\mu }}}e^{\frac{\left(-x\right)}{\beta_{\mu }}},\alpha_{\mu }>0,\beta_{\mu }>0,$$

where Γ(x) is a Gamma function. This Gamma function is defined as $\Gamma \left(x\right)=\int_{0}^{\infty }f^{x}-1\;e^{-f}df$, which is a definite integral for ℜ[x].

The parameters of the Gamma Mixture Model, defined in Equation (1), are solved by maximizing the log likelihood function, which is defined as

(3) $$\zeta \left(\theta |X_{d}\right)=\sum_{i=1}^{N}log\left(\sum_{\mu =1}^{M}\pi_{\mu }g\left(x_{i}|\alpha_{\mu },\beta_{\mu }\right)\right),$$

This Equation (3) is solved using the Expectation Maximization algorithm [43].

The GMMs generated with different numbers of components and solved by Expectation Maximization algorithm, the best-fitted GMM is selected by using second-order variant of AICc (Akaike Information Criterion) [44]. AICc is defined as

(4) $$AICc=-2\log\left(\zeta \left(\theta^{*}\right)\right)+2Y\left(\frac{N}{N-Y-1}\right),$$

where *N* is the total number of distances in $X_{d}$, Y is the number of parameters, and $\zeta \left(\theta^{*}\right)$ is the maximum log-likelihood value of the Gamma Mixture Model. The best fitted GMM is selected based on the the smallest value of AICc.

The dataset *X* is partitioned into M subsets based on the components of the best-fitted GMM, in which each subset is identified by one GMM component. As discussed in example, Figure 1c shows the vertical lines which partition the components, generated from the distance distribution, into four zones. The partitions of the four zones on the original data space is shown in Figure 1d. It is clearly seen from the Figure 1d that some component models identify more than one cluster, such as the model Z2 while some other component models identify one cluster, such as the models of Z1, Z3, and Z4.

The criteria to decide whether the subset of points needs further partitioning or not, are as follows:

1. If the number of objects in the subset is greater than the given threshold of minimum points, a new observation point is assigned to the data space, and the distance distribution vector is computed with respect to the observation point. If the distance distribution contains only one peak, the subset is made as a lead node. Otherwise, a new GMM is built from the distance distribution to further partition the subset.
2. In case of the number of objects in the subset is smaller than the threshold specified for the minimum number of points, the subset is made a leaf node without further partitioning.

**Figure 2:**
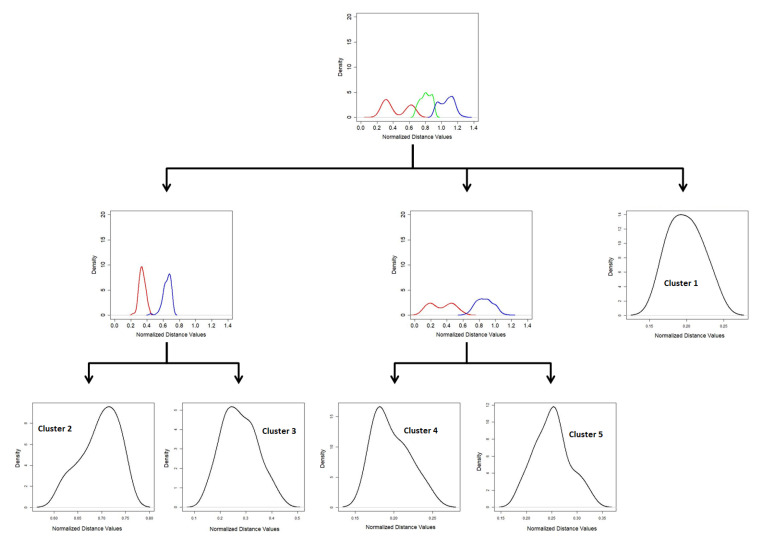
A GMM tree for identifying the number of clusters in a dataset.

The process continues until a GMM tree is built by generating all leaf nodes. An example of GMM-tree is shown in Figure 2.

Each leaf node of the GMM tree represents a cluster in the original dataset. A postprocessing step using the *k* nearest neighbor method is carried out on the set of objects in each leaf node due to the complexity of the dataset. The postprocessing step are discussed in detail in [35]. Our purpose is to illustrate the basic process of generating the GMM Tree. If a leaf node of the GMM Tree does not have high density due to less number of objects, then the objects are considered as outliers and that leaf node is ignored. In case of more than one dense region in a leaf node, the centroid objects of each dense region are taken as initial cluster centers. The number of initial cluster centers is considered as the number of cluster candidates which is the output of the GMM Tree algorithm. The number of clusters and the initial cluster centers found by the GMM Tree algorithm was used as an input parameter to the clustering algorithm used in our proposed method to find decision clusters which were used to classify the dataset.

## 4. Stratified Subspace Sampling GMM-Based Method (SSS-GMM)

This section presents a novel ensemble method to classify high-dimensional complex data with a large number of classes. In this method, we first cluster the data by using the ensemble clustering approach, and then we train the ensemble clustering result to make decision clusters by assigning the class labels to clusters based on the dominant class. These decision clusters are used to classify the new objects by computing the distances between the new objects and the centers of the decision clusters. The class label of the decision cluster is assigned to the new objects, which has a minimum distance from the center of the decision cluster. This ensemble method is named the stratified subspace sampling GMM-based method (SSS-GMM). In the following subsections, we present the key steps of SSS-GMM in detail.

### 4.1. Generation of Feature Strata from the Training Dataset $D_{train}$

Given a high-dimensional training dataset $D_{train}$, we first partitioned the features of $D_{train}$ into *L* feature clusters/strata in such a way that the features belonging to the same stratum were highly correlated. To find these closely related feature strata, we used k-means. As k-means requires the number of feature strata *L* as an input parameter, the GMM Tree method was used on the features of $D_{train}$ to discover the number of feature clusters *L* (or feature strata) and initial feature cluster centers. This *L* number of feature cluster centers were given to k-means as an input parameter, and the k-means algorithm generated feature clusters by grouping the features of $D_{train}$ into *L* feature strata $\left\{F_{1},F_{2},\ldots ,F_{L}\right\}$ based on correlation. This process is discussed in detail in [45].

In the case of a large number of objects in $D_{train}$, a percentage of objects ϑ was selected from $D_{train}$ and the GMM Tree algorithm was used to estimate the number of feature strata $\iota_{i}$. This process of selecting ϑ percent of objects and finding the number of clusters was repeated ν times. The number of feature strata *L* was computed by Max($\iota_{1}$,$\iota_{2}$, …, $\iota_{\nu }$), which was given to the k-means algorithm to partition the features of $D_{train}$ into *L* feature strata $\left\{F_{1},F_{2},\ldots ,F_{L}\right\}$.

### 4.2. Generation of Subspace Data Sets from the Training Dataset $D_{train}$

After generation of the feature strata $\left\{F_{1},F_{2},\ldots ,F_{L}\right\}$, subspace data sets were generated by stratified sampling without replacement, as follows. To generate *T* subspace data sets $\left\{D_{1},D_{2},\ldots ,D_{T}\right\}$ from $D_{train}$, $d_{\tau }\times \left(p_{l}/p\right)$ features were randomly selected without replacement from each feature stratum $F_{l}$, where $d_{\tau }$ is the number of features in subspace dataset $D_{\tau }$ for (1≤τ≤T), and $p_{l}/p$ is the proportion of features in $F_{l}$. These selected features from each stratum were combined into a single subset of $d_{\tau }$ features. This process was repeated T=p/d times, and *T* subsets of features were generated. After that, *T* subspace data sets $\left\{D_{1},D_{2},\ldots ,D_{T}\right\}$ were generated by extracting the the corresponding data from $D_{train}$ for each feature’s subset in such a way that $D_{\tau }\cap D_{t}=\emptyset$ for (τ≠t) and $\cup_{\tau =1}^{T}D_{\tau }=D$.

### 4.3. Generation of Clustering Results from Subspace data sets

After generating the subspace data sets $\left\{D_{1},D_{2},\ldots ,D_{T}\right\}$, the next step was to partition each subspace dataset into clusters. For this purpose, the GMM Tree algorithm was used first to estimate the number of clusters and initial cluster centers in each subspace dataset, and then the k-means algorithm was used to partition each subspace dataset to generate the clustering result.

After producing the clustering results from each subspace dataset $D_{\tau }$, we needed to ensemble these base clustering results of subspace data sets into the global clustering results of $D_{train}$.

### 4.4. Generation of Ensemble Clusters from Individual Subspace Clustering Results

To generate an ensemble clustering result from the base clustering result $\Psi_{1},\Psi_{2},\ldots ,\Psi_{T}$, we used the link-based approach in [36] to ensemble the $\Psi_{\tau }$ into an object cluster association (OCA) matrix, where each base clustering result $\Psi_{\tau }=\left\{C_{1}^{\tau },C_{2}^{\tau },\ldots ,C_{k_{\tau }}^{\tau }\right\}$ represented the clustering result of the $\tau^{\mathrm{th}}$ subspace dataset. Each cluster $C_{i}^{\tau }$ contained a subset of objects in $D_{train}$ where $C_{i}\neq \emptyset$ for $\left(1\leq i\leq k_{\tau }\right)$, $\cup_{\tau =1}^{k_{\tau }}C_{i}=D_{train}$ and $C_{i}\cap C_{j}=\emptyset$ for (i≠j).

For any two base clusterings $\Psi_{i}$ and $\Psi_{j}$, the link $e_{x,y}$ between cluster $C_{x}\in \Psi_{i}$ and cluster $C_{y}\in \Psi_{j}$ was computed as

(5) $$e_{x,y}=\frac{|C_{x}\cap C_{y}|}{|C_{x}\cup C_{y}|},$$

where $1\leq x\leq k_{i}$ and $1\leq y\leq k_{j}$.

Two clusters $C_{x}\in \Psi_{i}$ and $C_{y}\in \Psi_{j}$ were linked if $e_{x,y}>0$. By computing these links between clusters in different base clustering results, the similarity was computed between two clusters of the same clustering result that is, $C_{x},C_{y}\in \Psi_{\tau }$ as

(6) $$SIM\left(C_{x},C_{y}\right)=\frac{WTQ_{x,y}}{WTQ_{max}}*\beta ,$$

where β∈[0,1] is a threshold for the confidence level of considering the two clusters as being similar, and $WTQ_{x,y}$ is called the Weighted Triple Quality that was used to measure the strength of the indirect connection between $C_{x}$ and $C_{y}$ through clusters in other clustering results which form triples with $C_{x}$ and $C_{y}$ through links. $WTQ_{x,y}$ is calculated as follows:

- Find all clusters in other clustering results which are directly linked to $C_{x}$ and $C_{y}$ to form triples. Assume the set of *k* clusters directly linked to $C_{x}$ and $C_{y}$ is $N_{k}$.
- For each cluster $C_{t}$ in $N_{k}$, let $W_{t}$ = $e_{t,x}+e_{t,y}$ and $WTQ_{x,y}^{t}=\frac{1}{W_{t}}$.
- For all clusters in $N_{k}$, $WTQ_{x,y}$ is calculated as
  (7) $$WTQ_{x,y}=\sum_{t=1}^{k}WTQ_{x,y}^{t}.$$

$WTQ_{max}$ is the maximum value of all $WTQ_{x,y}$.

Given a clustering result $\Psi_{\tau }$, the association of each object $x_{i}$ in *D* to the clusters $\left\{C_{1}^{\tau },C_{2}^{\tau },\ldots ,C_{k_{\tau }}^{\tau }\right\}$ is calculated as

(8) $$OCA\left(x_{i},C_{t}\right)=\left\{\begin{array}{ll} 1 & \mathrm{if}C_{t}=C^{*}\left(x_{i}\right)\\ \mathrm{SIM}(C_{t},C^{*}\left(x_{i}\right)) & \mathrm{otherwise} \end{array}\right.$$

where $C^{*}\left(x_{i}\right)$ is the cluster to which $x_{i}$ belongs, and SIM($C_{t},C^{*}\left(x_{i}\right)$) ∈ [0,1] is calculated in Equation (6).

By computing the cluster associations of all clusters in $\Psi_{1},\Psi_{2},\ldots ,\Psi_{\tau }$ and all objects in $D_{train}$ with Equation (8), a new object cluster association matrix (OCA matrix for short) was obtained as a new representation of $D_{train}$. In the OCA matrix, each row indicated an object in $D_{train}$, while each column was a cluster in one of the τ base clustering results $\Psi_{1},\Psi_{2},\ldots ,\Psi_{\tau }$. Thus, each element in the OCA matrix indicates the association level of the row object to the column cluster. This computed OCA matrix is the new representation of dataset $D_{train}$ which was used to compute the ensemble clustering result. For this purpose, we first ran the GMM Tree algorithm again on an OCA matrix to find the number of clusters and initial cluster centers, and then used them as inputs to k-means to cluster the OCA and produce the final ensemble clustering result.

### 4.5. Assignment of Class Labels to the Clusters in Ensemble Clustering Result

After producing the ensemble clustering result, the class labels were assigned to the clusters as follows:

- The purity of each cluster in the ensemble clustering result, produced by k-means in the previous step, were computed based on the percentage of each class.
- The dominant class label was assigned to each cluster after computing the purity.
- As we only needed the clusters which were pure, we kept the clusters in which objects belonging to the dominant class were greater than threshold α percent. The clusters in which dominant class objects were less than the threshold α were discarded.

### 4.6. Classification of the Objects in the Testing Dataset $D_{test}$ Based on Generated Classifier

After assigning the class labels to the clusters in the previous step, the center of each cluster was computed by finding the mean object. This clustering result with an assignment of the dominant class was used as a classifier to classify new objects as follows:

- The distances between objects of the testing dataset $D_{test}$ and the center of each cluster in the classifier were computed.
- The class label of the cluster to the objects of $D_{test}$ based on the shortest distance was assigned.

**Figure 3:**
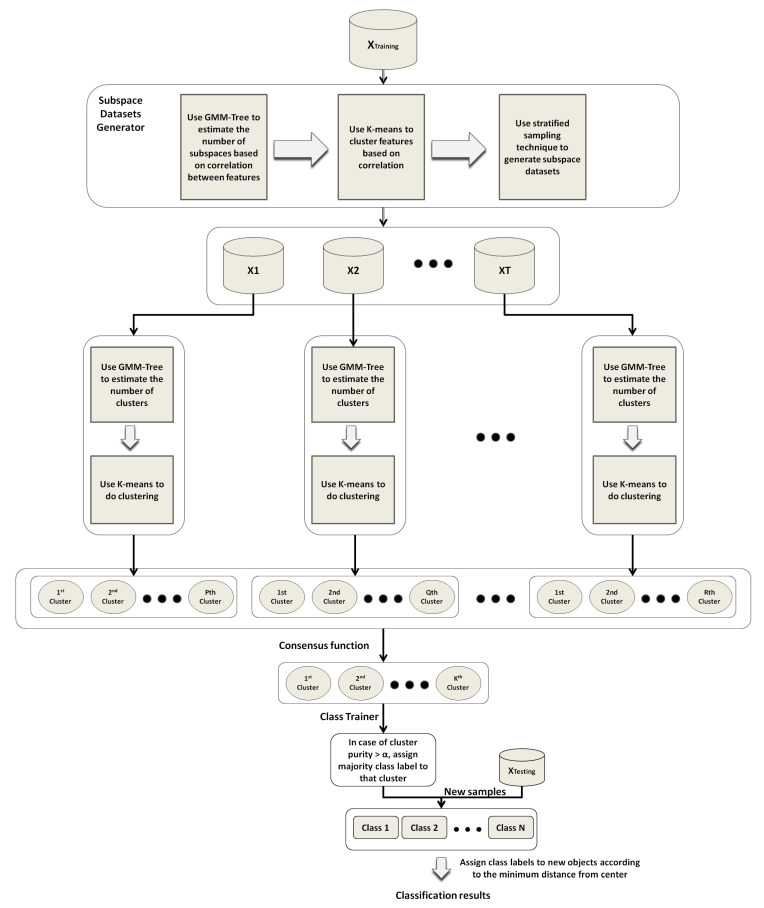
The process of SSS-GMM to classify a high-dimensional dataset.

The steps of the SSS-GMM are summarized in Figure 3.

The pseudo code was given in Algorithm 1, which took a high-dimensional training dataset $D_{train}=\left(x_{1},x_{2},\ldots ,x_{n}\right)$, test dataset $D_{test}$, number of iterations ν to find feature strata, percentage of objects μ to estimate the number of feature strata *L*, and the number of subspace data sets T as inputs, whereas its output was the classification accuracy on the test set $D_{test}$.

**Algorithm 1:** SSS-GMM algorithm

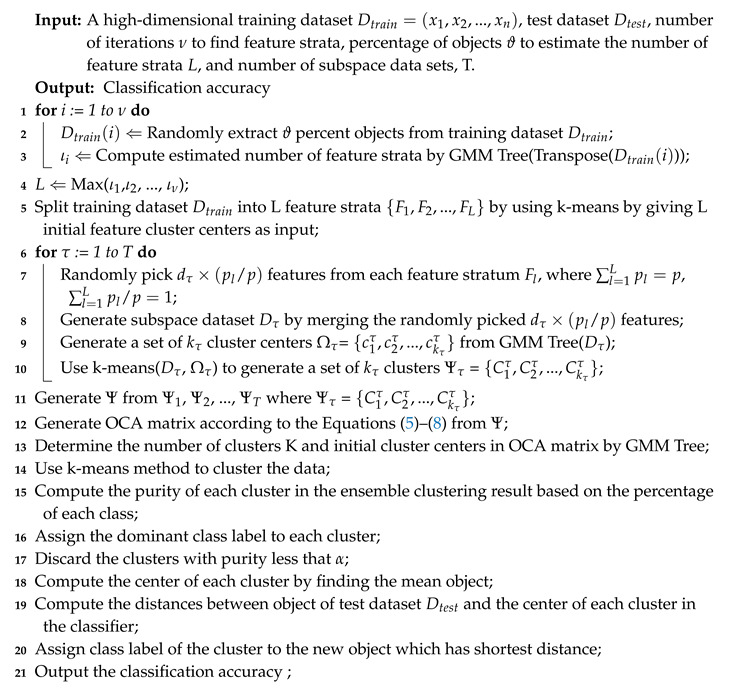



## 5. Experiments

In this section, we present the performance analysis of SSS-GMM in comparison with k-NN, Bagging, C4.5, Random Forest, and Adaboost in terms of accuracy and computational time. We have used both synthetic and the real-world data sets in the experiments to demonstrate the performance of our proposed method. These synthetic and real-world data sets are diverse in terms of the number of features (p), objects (N), and classes (C) which are helpful to analyze the performance of our proposed SSS-GMM. First, the characteristics of the synthetic and real-world data sets are described. Then, the experimental settings are discussed. Finally, the experimental results of SSS-GMM are discussed in comparison with the other state-of-the-art methods in terms of accuracy. Since our proposed SSS-GMM relies on the construction of a decision cluster tree, which is, to some extent, like a decision tree, it would be more convincing to compare our method with state-of-the-art decision tree-like algorithms, including Bagging, C4.5, Random Forest, and Adaboost. In addition to this, we have compared our proposed method with k-NN because we have also used k-NN in building our proposed classifier.

### 5.1. Data Sets

The experiments were conducted on 12 synthetic data sets and eight real-world data sets. R package clusterGeneration [46] was used to generate the 12 synthetic data sets. The characteristics of these synthetic data sets are listed in Table 1. The first column from the left shows the dataset name. The second column describes the number of objects N in each dataset. The third and the fourth columns show the number of features p and the number of clusters *K* given in each dataset, respectively. The same value of cluster separation index = 0.5 was used for each synthetic dataset which indicated the degree of separation between a cluster and its nearest neighboring cluster. We used this constant value for each synthetic dataset to see the trend change in the accuracy based on the change in the number of features and the number of classes. Each cluster contained 100 objects following a normal distribution. Thus, to generate the synthetic data sets with the settings according to Table 1, we set parameters of clusterGeneration as sepVal (cluster separation index) = 0.5, numClust (Number of clusters) according to Table 1: column 4 (Classes), clustSizeEq (cluster objects size) = 100, numNonNoisy (Number of features) according to Table 1: column 3 (features).

To evaluate the performance of our proposed method on real data, eight real-world data sets were selected. The details of the real-world data sets have been summarized in Table 2. These data sets were high-dimensional, and the features ranged from 64 to 8460, taken from the UCI [47] and KEEL repository [48]. All data sets were multiclass, with a range of 10 to 983. Optdigits was the Optical Recognition of Handwritten Digits Data Set. The fbis dataset was from the Foreign Broadcast Information Service data of TREC-5. Wap is a skew dataset with 20 classes. The remaining data sets were selected from the KEEL dataset repository, while the Aloi(short) dataset was taken from Amsterdam Library of Object Images dataset repository [49]. The Aloi(short) dataset contained 10,800 objects representing 100 objects (clusters). These data sets had diverse representations in application domains and the numbers of objects, features, and classes.

The class labels were used to train our proposed method during the assignment of class labels to generated clusters. These class labels were also used as ground truth to compare performance accuracy on the testing set. The objects in some of these data sets belonged to more than one class. Thus, classifying these kinds of data sets was more difficult due to high overlapping.

### 5.2. Experimental Settings

Here, we show the comparison results of SSS-GMM and other five classification methods: decision tree (C4.5), Bagging, Original k-NN, Random Forest, and Adaboost on eight real data sets. In our experiments, we used default parameters for C4.5, Random Forest, and k-NN in R. For C4.5, the minimum number of instances per leaf was set as 2. For Random Forest, the random number seed was set to 1. For k-NN, the number of neighbors, k, equaled to 1. The base classifier to be used in Bagging and AdaBoost was C4.5. The default values for the GMM Tree algorithm (used in our proposed method) were set according to [35]. The package mixtools [50] was used for GMM and EM. For SSS-GMM, we set μ as 20 percent and the number of iterations ν as 10. In this way, 20 percent of objects were randomly extracted 10 times to find the number of feature strata, and then the maximum of these 10 iterations was used as the initial estimation of the number of feature strata in the dataset *D*. The threshold parameter α was set as 90 percent to set the purity level of each cluster. We used the 10-fold method to get classification accuracy. The number of subspace data sets M was set as 10, which can be changed by the user but should not be very high because it will generate more GMM Trees and increase the computation cost. The value of β used in Equation (6) was set to 0.8 as the default value [36]. The minimum number of objects was set to be 10 as a termination threshold for a leaf node. If a subset has 10 objects or less, this subset is either a small cluster or a set of outliers. There is no need to partition it further.

The experiments were conducted on machines with 128 GB memory running on Windows Server 2012 R2 Standard and 3.30 GHz Intel(R) Core(TM)i5-4590 CPU with 12 GB of memory running Windows 7 of 64-bit.

### 5.3. Experimental Results

**Figure 4:**
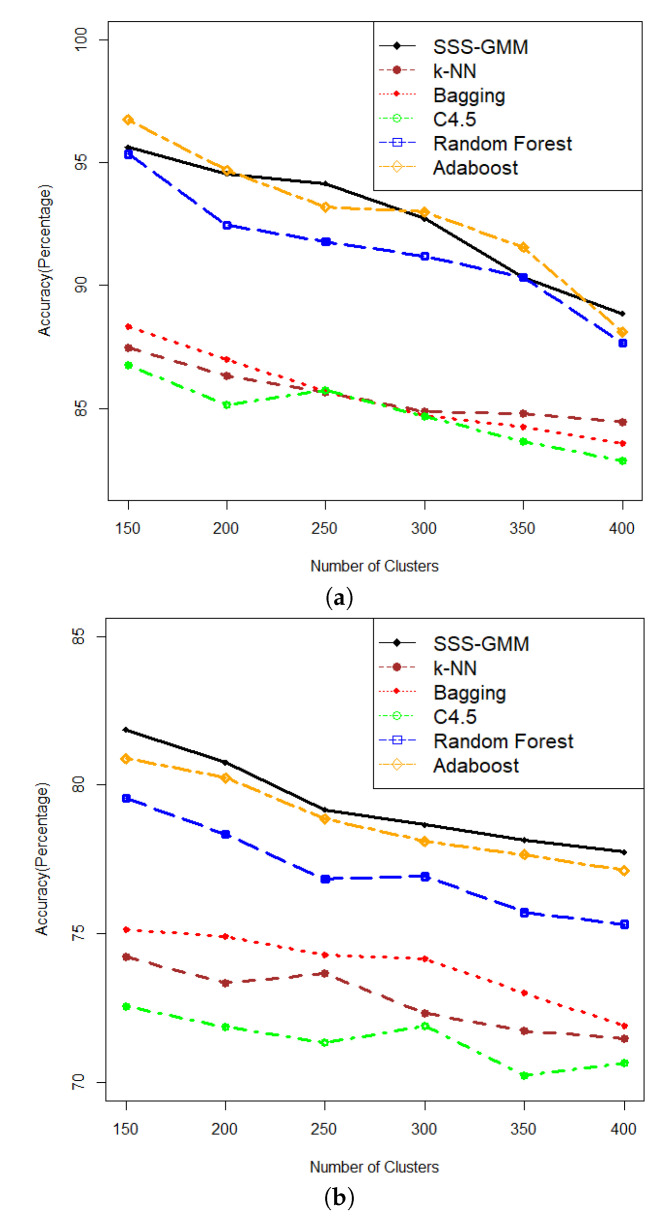
Accuracy comparison on synthetic data sets (SSS-GMM, k-NN, Bagging, C4.5, Random Forest, Adaboost). (**a**) Synthetic data sets (DS1-DS6) Features = 200 and Objects (15,000–40,000). (**b**) Synthetic data sets (DS7-DS12) Features = 1000 and Objects (15,000–40,000).

In this section, we discuss the results on the comparison of our proposed algorithm with other methods in classification accuracy. The comparative results of SSS-GMM and other classification methods on synthetic data sets are given in Table 3. We can see that on comparatively low-dimensional datasets, that is, from DS1-DS6, our proposed SSS-GMM performed better than C4.5, Bagging, Original k-NN, and Random Forest, except Adaboost. While on high-dimensional data sets, that is, from DS7-DS12, our method outperformed all other methods. As a whole, our proposed SSS-GMM achieved the best performance in eight data sets out of 12 synthetic data sets, and was only slightly worse than the best method in other four data sets. In general, our proposed SSS-GMM was the best-performing method, followed by Adaboost. The accuracy performance is shown in Figure 4 where Figure 4a shows the accuracy of all methods on data sets DS1-DS6 and Figure 4b shows the accuracy on data sets DS7-DS12.

**Figure 5:**
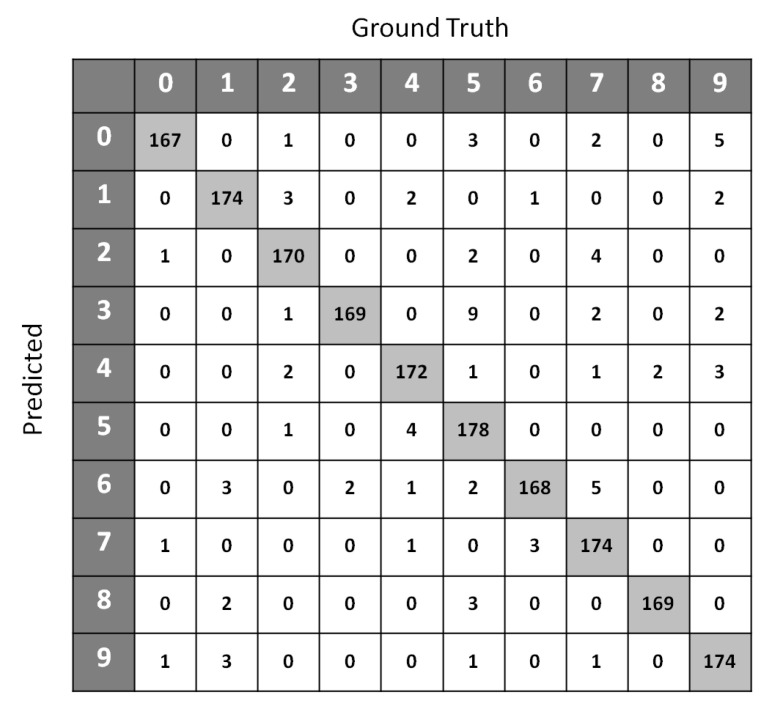
Confusion matrix of Optdigits (test set) generated by SSS-GMM.

**Figure 6:**
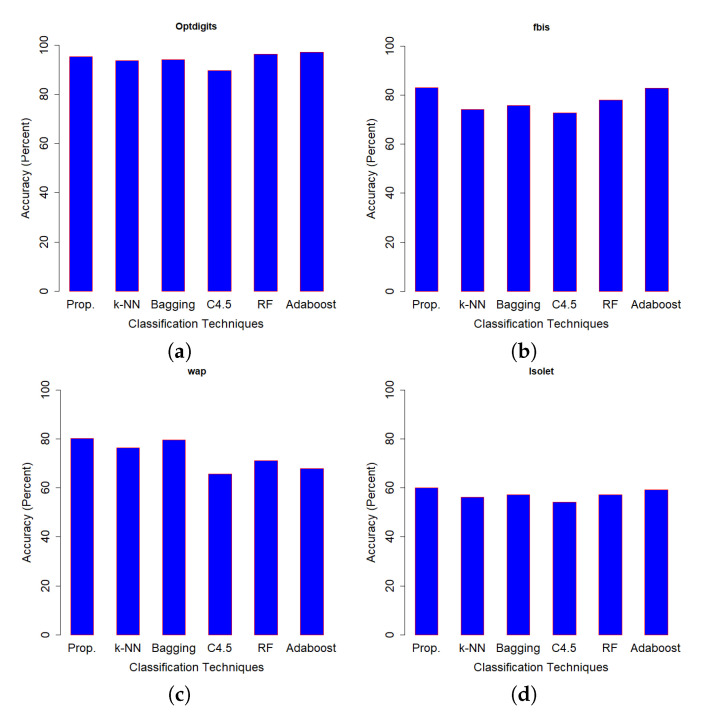
Accuracy comparison (Prop = SSS-GMM, k-NN, Bagging, C4.5, RF = Random Forest, Adaboost) data sets: (**a**) Optdigits, (**b**) fbis, (**c**) wap, (**d**) Isolet.

**Figure 7:**
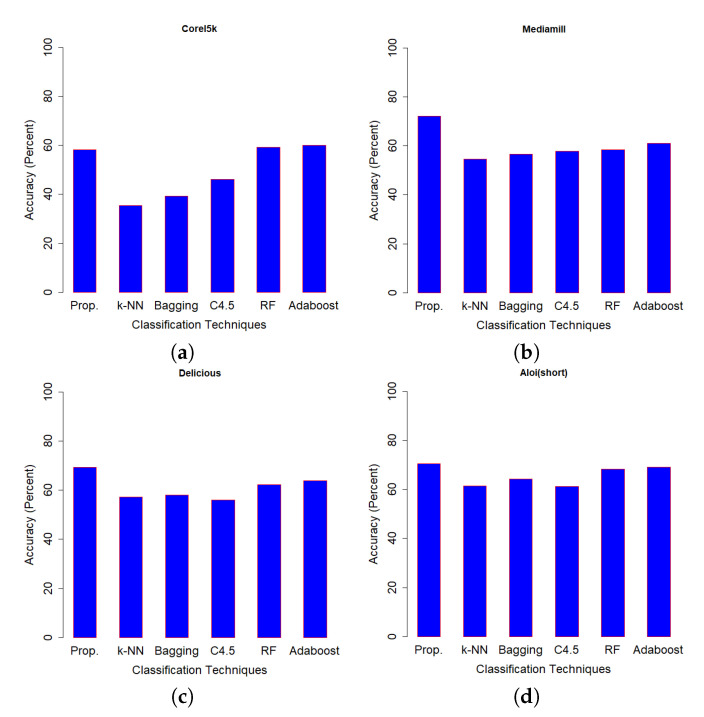
Accuracy comparison (Prop = SSS-GMM, k-NN, Bagging, C4.5, RF = Random Forest, Adaboost) data sets: (**a**) Corel5k, (**b**)Mediamill, (**c**) Delicious, (**d**) Aloi(short).

Table 4 shows the classification accuracy performance of SSS-GMM and other classification methods on real data sets. A confusion matrix of Optdigits(test set) generated by SSS-GMM is shown in Figure 5 which was used to find the classification accuracy on the test set of Optdigits dataset. We can see that, except for two data sets (Optdigits and Corel5k), our proposed SSS-GMM outperformed all methods. As a whole, our proposed SSS-GMM achieved the best performance in six real-world data sets out of eight data sets. Thus, our proposed SSS-GMM was also the best-performing method, followed by Adaboost on real-world data sets. Adaboost is a state-of-the-art method which can handle low-dimensional data without noise. However, in the case of high-dimensional noisy data and outliers, its performance deteriorates. Thus, in the case of most of synthetic data sets, the performance of Adaboost and SSS-GMM is similar, but in the case of real data sets, our proposed SSS-GMM performed better. The accuracy performance on various data sets is shown in Figure 6 and Figure 7. Figure 6 shows the classification accuracy on the data sets which had a relatively less number of classes (i.e., 10–26 classes) as compared to the data sets used in Figure 7 where all data sets have more than 100 classes.

Table 5 shows the time comparison of SSS-GMM with five other state-of-the-art methods on DS7-DS12. We selected these data sets due to their high dimensions and large number of classes. From the results, it is clear that the testing time of SSS-GMM is at a minimum as compared to other state-of-the art methods, which can compensate for the shortcoming of SSS-GMM of taking time during training. The testing time of SSS-GMM was shortest because it only needs to find the distances between centers of the clusters and the objects of the testing data sets. Thus, overall, our proposed SSS-GMM outperformed other methods in terms of accuracy and testing time.

To validate the clustering result, we investigated the quality of the clustering results. To measure the clusters’ quality, we used ARI [51] and Purity [52]. The Purity was defined as:

(9) $$Purity=\frac{1}{N}\sum_{k=1}^{K}max_{1\leq q\leq \bar{K}}\left|C_{k}\cap Y_{q}\right|$$

where *K* is the number of true clusters, $C_{k}$ is the set of objects in true cluster *k*, $\bar{K}$ is the number of clusters by k-means, *N* is the total number of objects in the dataset, and $Y_{q}$ is the set of objects in cluster *q* by k-means. $max_{1\leq q\leq \bar{K}}$ denotes that for $\bar{K}$ clusters by k-means, only the cluster *q* whose intersection with $C_{k}$ has the largest number of objects is considered. |.| indicates the number of objects in the intersection of $C_{k}$ and $Y_{q}$. The value of Purity is between 0 to 1.

ARI [51] was also used to measure the clustering performance. It is a measure of agreement between two partitions: one given by the clustering process, and the other defined by the ground truth. Given a dataset *D* of *N* objects, and two partitions of these *N* objects, namely, $C=C_{1},C_{2},\ldots ,C_{K}$ being a partition of *N* objects into *K* clusters and $Y=Y_{1},Y_{2},\ldots ,Y_{P}$ being a partition of *N* objects into *P* classes (the ground truth), ARI is defined as:

(10) ARI=∑jkNjk2−∑jαj2∑kβk2/N21/2∑jαj2∑kβk2−∑jαj2∑kβk2/N2

where $N_{\mathrm{jk}}$ is the number of objects in cluster $C_{j}$ and the second partition, $Y_{k}$, $\alpha_{j}$ is the number of objects in the first partition, $C_{j}$, and $\beta_{k}$ is the number of objects in the second partition, $Y_{k}$.

The Purity and ARI results on synthetic data sets are shown in Table 6, while the Purity and ARI values measured by SSS-GMM on real data sets are shown in Table 7. From the results, it is clearly seen that SSS-GMM performed very well in finding clusters on synthetic data sets, while in the case of real data sets, it also performed well.

Finally, we have also shown the number of clusters found during construction of the SSS-GMM for validation purposes. As shown in Table 8 and Table 9, we found that our proposed SSS-GMM successfully discovered most of the classes in both synthetic and real-world data sets.

From the above experimental results, we can conclude that our algorithm performed better than all other methods on most of the data sets which were diverse in nature in terms of the number of features and the number of classes.

## 6. Conclusions and Future Work

In this paper, we proposed a new hierarchical Gamma Mixture Model-based method (named SSS-GMM) for classifying high-dimensional data with a large number of classes which used a subspace ensemble approach to deal with this challenging problem by integrating multiple techniques in an innovative way. For this purpose, we first used the GMM Tree to find the number of feature strata, and then a k-means algorithm to divide the set of features of the dataset into feature strata. Then, the stratified subspace sampling method was used to sample subspace features from the feature strata and generate a set of subspace data sets from the high-dimensional dataset. After that, the GMM Tree algorithm was used again to identify the number of clusters and initial clusters in each subspace dataset for the k-means algorithm to cluster the subspace dataset. Then, the link-based method was used to integrate the subspace clustering results into an object cluster association matrix, from which the ensemble clustering result was generated by the k-means algorithm with the number of clusters identified by the GMM Tree algorithm. After producing the ensemble clustering result, the dominant class label was assigned to each cluster after computing the purity. A classification was made on the object by computing the distances between the new object and the center of each cluster in the classifier, and the class label of the cluster was assigned to the new object which had the shortest distance. A series of experiments were conducted on twelve synthetic and eight real-world data sets with different numbers of classes, features, and objects. The experimental results have shown that the new method performs better in classifying data in all data sets as compared to the other state-of-the-art techniques.

Our future work consists of analysing SSS-GMM on noisy data with thousands of features and classes. We will also analyse the performance of this method on high-dimensional regression data sets.

## Figures and Tables

**Table 1:** Characteristics of the synthetic data sets.

Data Sets	Objects (N)	Features (p)	Classes (C)
DS1	15,000	200	150
DS2	20,000	200	200
DS3	25,000	200	250
DS4	30,000	200	300
DS5	35,000	200	350
DS6	40,000	200	400
DS7	15,000	1000	150
DS8	20,000	1000	200
DS9	25,000	1000	250
DS10	30,000	1000	300
DS11	35,000	1000	350
DS12	40,000	1000	400

**Table 2:** Characteristics of the real-world data sets.

Number	Data Sets	Objects	Features	Classes
1	Optdigits	5620	64	10
2	fbis	2463	2000	17
3	wap	1560	8460	20
4	Isolet	6238	617	26
5	Aloi(short)	10,800	128	100
6	Corel5k	5000	499	374
7	Mediamill	43,907	120	101
8	Delicious	16,150	500	983

**Table 3:** Synthetic data sets: Comparison of SSS-GMM with other five state-of-the-art methods in terms of accuracy.

Data Sets	SSS-GMM	k-NN	Bagging	C4.5	Random Forest	Adaboost
DS1	95.61	87.45	88.34	86.76	95.34	**96.73**
DS2	94.54	86.34	86.98	85.15	92.45	**94.67**
DS3	**94.15**	85.67	85.71	85.74	91.77	93.18
DS4	92.72	84.86	84.73	84.67	91.19	**92.99**
DS5	90.34	84.78	84.23	83.66	90.32	**91.56**
DS6	**88.85**	84.45	83.56	82.87	87.67	88.11
DS7	**81.85**	74.23	75.14	72.56	79.56	80.89
DS8	**80.76**	73.34	74.92	71.87	78.34	80.23
DS9	**79.17**	73.67	74.29	71.34	76.84	78.86
DS10	**78.67**	72.34	74.15	71.89	76.93	78.11
DS11	**78.15**	71.74	73.02	70.23	75.72	77.67
DS12	**77.75**	71.47	71.89	70.67	75.32	77.13

**Table 4:** Real data sets: Comparison of SSS-GMM with other five state-of-the-art methods in terms of accuracy.

Data Sets	SSS-GMM	k-NN	Bagging	C4.5	Random Forest	Adaboost
Optdigits	95.43	93.72	94.11	89.72	96.42	**97.20**
fbis	**82.99**	74.23	75.82	72.84	78.03	82.92
wap	**80.23**	76.41	79.63	65.64	71.15	67.95
Isolet	**60.12**	56.21	57.21	54.21	57.21	59.21
Aloi(short)	**70.51**	61.43	64.32	61.22	68.34	69.13
Corel5k	58.23	35.45	39.25	46.21	59.21	**59.99**
Mediamill	**72.14**	54.66	56.65	57.81	58.45	61.13
Delicious	**69.32**	57.34	57.99	56.13	62.36	63.83

**Table 5:** Synthetic data sets: Training/testing time comparison of SSS-GMM with five other state-of-the-art methods (time is in Minutes:Seconds format).

Data Sets	SSS-GMM	K-NN	C4.5	Bagging	Random Forest	Adaboost
DS7	05:24/00:16	00:00/02:13	02:02/00:33	05:33/00:33	04:12/01:02	02:51/00:21
DS8	06:01/00:19	00:00/02:32	02:09/00:48	06:13/00:39	04:53/01:14	03:13/00:29
DS9	06:42/00:21	00:00/02:51	02:22/00:59	07:03/00:44	05:24/01:23	03:36/00:35
DS10	07:33/00:24	00:00/03:11	02:29/01:13	07:41/00:49	05:56/01:29	03:54/00:43
DS11	07:51/00:29	00:00/03:23	02:36/01:41	08:32/00:54	06:11/01:36	04:23/00:56
DS12	08:21/00:37	00:00/03:49	02:48/01:49	08:58/00:59	06:42/01:45	04:52/01:11

**Table 6:** Purity and ARI measured by SSS-GMM on synthetic data sets.

Data Sets	Classes	Purity	ARI
DS1	150	0.896	0.866
DS2	200	0.887	0.857
DS3	250	0.876	0.856
DS4	300	0.868	0.845
DS5	350	0.865	0.849
DS6	400	0.854	0.838
DS7	150	0.797	0.768
DS8	200	0.794	0.762
DS9	250	0.783	0.744
DS10	300	0.787	0.739
DS11	350	0.778	0.728
DS12	400	0.776	0.734

**Table 7:** Purity and ARI measured by SSS-GMM on real data sets.

Data Sets	Classes	Purity	ARI
ISOLET	26	0.601	0.614
Aloi(short)	100	0.718	0.696
Corel5k	374	0.636	0.612
Mediamill	101	0.616	0.592
Delicious	983	0.706	0.681
Bibtex	159	0.528	0.532
Bookmarks	208	0.514	0.547
Topics	101	0.583	0.594
Industries	313	0.534	0.511
Regions	228	0.519	0.513

**Table 8:** Clusters found in the synthetic data sets by SSS-GMM.

Dataset	Actual Clusters	Total Clusters Found	Unique Clusters Found
DS1	150	148	148
DS2	200	194	194
DS3	250	244	242
DS4	300	294	291
DS5	350	345	341
DS6	400	395	391
DS7	150	147	147
DS8	200	194	193
DS9	250	243	241
DS10	300	294	291
DS11	350	344	342
DS12	400	394	392

**Table 9:** Clusters found in the real-world data sets by SSS-GMM.

Number	Dataset	Classes	Total Clusters Found	Unique Clusters Found
1	Optdigits	10	14	10
2	fbis	17	20	17
3	wap	20	24	20
4	Isolet	26	39	26
5	Aloi(short)	100	128	97
6	Corel5k	374	423	368
7	Mediamill	101	121	97
8	Delicious	983	1012	977
